# Explainable Precision Medicine in Breast MRI: A Combined Radiomics and Deep Learning Approach for the Classification of Contrast Agent Uptake

**DOI:** 10.3390/bioengineering11060556

**Published:** 2024-05-31

**Authors:** Sylwia Nowakowska, Karol Borkowski, Carlotta Ruppert, Patryk Hejduk, Alexander Ciritsis, Anna Landsmann, Magda Marcon, Nicole Berger, Andreas Boss, Cristina Rossi

**Affiliations:** 1 Diagnostic and Interventional Radiology, University Hospital Zürich, University Zürich, Rämistrasse 100, 8091 Zürich, Switzerlandcristina.rossi@usz.ch (C.R.); 2 b-rayZ AG, Wagistrasse 21, 8952 Schlieren, Switzerland

**Keywords:** breast cancer risk, breast dynamic contrast-enhanced MRI, background parenchymal enhancement, BI-RADS-compliant BPE classification, deep neural networks, radiomics, explainable AI, Shapley values

## Abstract

In DCE-MRI, the degree of contrast uptake in normal fibroglandular tissue, i.e., background parenchymal enhancement (BPE), is a crucial biomarker linked to breast cancer risk and treatment outcome. In accordance with the Breast Imaging Reporting & Data System (BI-RADS), it should be visually classified into four classes. The susceptibility of such an assessment to inter-reader variability highlights the urgent need for a standardized classification algorithm. In this retrospective study, the first post-contrast subtraction images for 27 healthy female subjects were included. The BPE was classified slice-wise by two expert radiologists. The extraction of radiomic features from segmented BPE was followed by dataset splitting and dimensionality reduction. The latent representations were then utilized as inputs to a deep neural network classifying BPE into BI-RADS classes. The network’s predictions were elucidated at the radiomic feature level with Shapley values. The deep neural network achieved a BPE classification accuracy of 84 ± 2% (*p*-value < 0.00001). Most of the misclassifications involved adjacent classes. Different radiomic features were decisive for the prediction of each BPE class underlying the complexity of the decision boundaries. A highly precise and explainable pipeline for BPE classification was achieved without user- or algorithm-dependent radiomic feature selection.

## 1. Introduction

Magnetic Resonance Imaging (MRI) with Dynamic Contrast Enhancement (DCE) is a key imaging modality in breast diagnostics. It is indicated for opportunistic screening in female patients at high risk, for diagnostics, treatment planning, and monitoring, as well as for the treatment outcome assessment [1,2,3,4,5]. The European Society of Breast Imaging has recently updated the recommendation guidelines indicating that female patients with extremely dense breast tissue should undergo screening DCE-MRI examination on a regular basis [6], adding a further area of indications for this diagnostic approach.

During the DCE-MRI examination, a contrast agent is injected and its uptake by the normal fibroglandular tissue (FGT), i.e., Background Parenchymal Enhancement (BPE), may occur. BPE is an essential biomarker in precision medicine, as it is suggested to be correlated with breast cancer risk, as well as with breast cancer treatment outcome [7,8]. Moreover, it also can affect diagnostic accuracy by covering lesions (false negative) or being mistaken for them (false positive) [9]. Therefore, according to the current Breast Imaging Reporting & Data System (BI-RADS) atlas developed by the American College of Radiology, both FGT and BPE should be visually classified into four categories [10]. For FGT, these categories are as follows: *almost entirely fat*, *scattered*, *heterogeneous*, and *extreme*, while for BPE they are *minimal*, *mild*, *moderate*, and *marked*. It is essential to specify these categories in the radiological report to assist in decision-making for a given patient. The reported inter-reader agreements for the visual classification of BPE range from fair (Cohen’s kappa κ = 0.28 [11], κ = 0.36 [12]) via substantial (κ = 0.61 [13], κ = 0.78 [14]) to almost perfect (κ = 0.83 [15], κ = 0.93 [16]).

In our previous work [17], we developed FGT and BPE deep learning segmentation models and correlated the obtained volumetric measures with the classes assigned by the radiologists. Our results suggested that a standardized assessment of FGT can be solely based on volumetric measures, whereas in the case of BPE an additional model taking into account voxels’ intensity distribution and morphology is needed for a reliable classification.

Radiomics has shown potential in several breast cancer applications, including in distinguishing benign from malignant lesions, predicting axillary lymph node status, identifying molecular subtypes, predicting chemotherapy response, and estimating survival outcomes [18,19]. Radiomics was also used in the context of BPE assessment, in which the radiomics features were extracted from segmented contrast uptake and served as an input for a tree-based classification model [13]. Additionally, deep learning architectures have been employed for both two-class [20] and four-class BI-RADS compliant classifications, in which the direct classification of MRI images was performed by a Convolutional Neural Network (CNN) [14]. Those approaches demonstrated the suitability of radiomics and deep learning tools for the BPE classification task. However, higher classification accuracy is needed for a full integration into the clinical routine.

Building upon these insights, the main objective of this study was to develop an automated algorithmic pipeline for a (i) highly accurate and (ii) explainable BI-RADS-compliant BPE classification by combining the strengths of radiomics and deep learning.

## 2. Materials and Methods

### 2.1. MRI Datasets

This retrospective study was approved by the local ethics committee. The dataset was curated by searching in the Picture Archiving and Communication System (PACS) of our institution through examinations acquired between September 2013 and October 2015. The inclusion criteria were the following: (a) age above 18 years, (b) absence of implants, and (c) lack of artefacts. The resulting dataset was included in a prior article evaluating the feasibility of obtaining BPE class directly from MRI slices with the use of CNN [14]. The resulting model had an overall accuracy of 75%. However, it presented a relatively high misclassification rate between *moderate* and *mild* BPE class. In this work, we aimed to evaluate a different approach based on radiomics and deep learning to improve the performance in the discrimination of each BPE class. In this study, we used a subset of the dataset consisting of the first post-contrast subtraction images from MRI-DCE sequences for 27 healthy female subjects, resulting in 2403 slices. The examinations were acquired with a 3.0 T Siemens scanner.

Additionally, an external test set was employed to validate the developed pipeline, which comprised a subset of the public EA1141 dataset containing abbreviated breast MRI data [21]. From this dataset, examinations performed with 3.0 T Siemens scanners of healthy female subjects (BI-RADS 1) were selected. Subsequently, two examinations per each BPE class, assigned volumetrically, were chosen, resulting in 1082 slices depicting breasts. 

For detailed information about MRI imaging parameters of both datasets, see the Supplementary Material.

The BPE was classified slice-wise by two consenting radiologists, each with more than five years of breast imaging experience. Their classification serves as a gold standard in this research.

### 2.2. Explainable BPE Classification Pipeline

**Figure 1:**
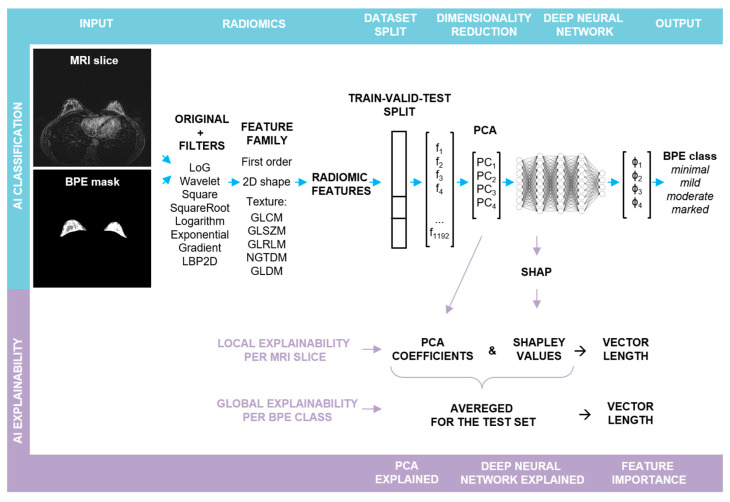
**Schematic representation of the major steps in the explainable BPE classification pipeline:** the upper row depicts the steps involved in the BPE classification, whereas the lower one illustrates the steps for obtaining local and global explainability of the feature importance. Abbreviations: Laplacian of Gaussian (LoG), Local Binary Pattern applied in 2D (LBP2D), Gray Level Cooccurrence Matrix (GLCM), Gray Level Size Zone Matrix (GLSZM), Gray Level Run Length Matrix (GLRLM), Neighboring Gray Tone Difference Matrix (NGTDM), Gray Level Dependence Matrix (GLDM).

The steps involved in the pipeline are illustrated in Figure 1 and described below:

(a) *BPE Segmentation.* The BPE was segmented in a semi-automatic way in a 3D slicer [22] using a grow from seeds algorithm as well as thresholding.
(b) *Radiomic feature extraction.* Prior to slice-wise feature extraction, the volumes were normalized. PyRadiomics v. 3.0.1 was used for the extraction of radiomic features [23]. The resampling was performed with a Bspline interpolator, [1, 1] resampled pixel spacing, a pad distance of 10 and enabled pre-crop. The bin width for discretization was set to 20 and the voxel array shift to 300. First post-contrast subtraction MRI images with corresponding BPE masks served as the input for the radiomic toolbox: first order, shape, and texture features were extracted from each slice in its original form, as well as from the same slice after the application of different filters (cf. Figure 1). For each slice, 1192 features were obtained.
(c) *Train–validation–test split.* In the first step, the dataset was split into two parts. The first part, containing approximately 80% of the data, was utilized for hyperparameter optimization through 5-fold cross-validation (CV). The optimized hyperparameters were then applied to train the final model, with the first part of the data serving as both the training and the validation sets (comprising 62% and 19% of slices, respectively) and the second part serving as an outer test set (comprising 19% of slices), cf. Figure S1. The initial split as well as the CV splits were performed randomly in a patient-stratified manner. As the distribution of the BPE classes in the dataset is imbalanced, the initial split was performed with the requirement that in each set, each BPE class had to be represented by a minimum of 10% slices with respect to all the slices contained in this set. For the cross validation, the requirement had to be lowered to a minimum of 2% slices.
(d) *Feature pre-processing and PCA.* Feature standardization followed by PCA was performed for each training set, with the validation and the test sets being transformed accordingly.
(e) *Deep Neural Network: training and evaluation.* A fully connected deep neural network (DNN) was built using sequential blocks consisting of dense layers, followed by batch normalization and dropout layers from the tensorflow.keras library. Hyperparameter tuning using different numbers of Principal Components (*PC*s) as input, different number of blocks with varying number of neurons and dropout rates, and different learning rates was performed (cf. Supporting Table S1). To ensure comparability, the random seed was fixed globally, and the weights were initialized with the use of the glorot uniform method and the biases at the value of zero. To compensate for the class imbalance, a weighted categorical cross-entropy loss was used. Each training run was performed for 150 epochs, and a model characterized by the lowest validation loss was chosen for the performance evaluation: confusion matrix and accuracy were calculated for the test set. For each parameter set, the mean and the standard deviation of accuracy of CV splits were calculated. Additionally, the confusion matrices were reviewed for false positive and false negative rates. The best parameter set was chosen, and the training was repeated for 10 runs without a fixed global random seed.
  Average accuracy and standard deviation were calculated for the resulting 10 models and *p*-value was computed using Student’s *t*-test (accuracy > 0.25). To evaluate a single model, bootstrap resampling with replacement was performed on the obtained predictions for a test set (n = 10,000). Accuracy and Cohen’s kappa (κ) scores were obtained for each resample and average values were calculated together with 95% confidence intervals (CI). *p*-values were determined utilizing Student’s *t*-test.
  The best performance was achieved for a deep neural network taking the first four *PC*s as input, with four blocks, each having 512, 256, 128, and 64 neurons in the dense layer, respectively. The dropout rate was equal to 0.45. The Adam optimizer with a learning rate of 0.0001 was applied. The training was performed in batches, each containing 50 samples.
(f) *Explainability.* From the trained final models, two were chosen for detailed analysis with a focus on the explainability and its robustness. The results obtained for one of them are presented in the main text (including an evaluation on the external test set), whereas for the second one the results can be found in the Supplementary Material. The next sections describe the analysis in detail.

### 2.3. Shapley Values

The initialization of the SHAP Kernel Explainer was performed with the trained DNN and the training set after PCA transformation [24]. In this way, base values for each BPE class were obtained. These correspond to “the average model output over the training dataset” [25]. Next, the explainer was applied to each slice from the test set. For a slice, four arrays with Shapley values were obtained, explaining the DNN output for each BPE class (*φ*_1_–*φ*_4_, in Figure 1). As the first four *PC*s served as an input to the DNN, each array consisted of four elements quantifying the impact of each *PC* on the given prediction.

### 2.4. Shapley-Scaled Vectors

Depending on the predicted class, a corresponding array with Shapley values was chosen. These served as scaling factors for PCA coefficients. Subsequently, the 4D Euclidean vector length in the PCA space for each radiomic feature was calculated. For the vector length calculation, the following equations were used:

(1) $${PC}_{j}=\sum_{i=1}^{n}\varphi_{ij}X_{i}$$

(2) $$\left|\vec{V_{Xi}}\right|=\sqrt{\sum_{j=1}^{4}\varphi_{i}^{\;2}}$$

(3) $$\left|\vec{V_{Xi\_SHAP}}\right|=\sqrt{\sum_{j=1}^{4}{\alpha_{j}\varphi }_{i}^{\;2}}$$

(4) $${\vec{V_{Xi\_SHAP}}}_{orientation}=\sum_{j=1}^{4}\alpha_{j}\varphi_{i}$$

$X_{i}—radiomic\;feature;$

${PC}_{j}—principal\;component;$

$\varphi_{ij}—coefficient\;associated\;with\;X_{i}\;and\;{PC}_{j};$

$\alpha_{j}—Shapley\;value\;for\;{PC}_{j}.$

Firstly, the contribution of each radiomic feature to each *PC* was retrieved from the PCA fit on the training set using Equation (1). The Euclidean vector’s length associated with each radiomic feature in 4D was calculated subsequently with the use of Equation (2). To assess the importance of each radiomic feature, the coefficients associated with each *PC* were multiplied by the corresponding Shapley value and served to calculate the Shapley-scaled 4D vector’s length for each feature (Equation (3)). In addition to the evaluation of radiomic feature importance by the length of the Shapley-scaled vector, the vector orientation in the feature space can be assessed by the summation of all Shapley-scaled coefficients (Equation (4)).

### 2.5. Local Explainability

Within the feature family (cf. Figure 1), one feature type can be extracted from a slice, not only after applying different filters but also after different mathematical transformations. For example, the Gray Level Non-Uniformity feature was extracted after calculating GLRLM, GLDM, and GLSZM matrices. Thus, to explain the DNN predictions, firstly, the length values of the Shapley-scaled vectors were sorted by the feature type together with the corresponding orientation values (cf. Figure S2a). As in most cases, the vector length and orientation values were similar within one feature type; in the next step, their averaging was performed, followed by scaling the length values to the [0, 1] range to facilitate the comparability (cf. Figure S2b). In the last step, all vector length values below the 75th percentile with the corresponding orientation values were excluded.

### 2.6. Global Explainability

For the global explainability, the test set was divided into four subsets according to the BPE class predicted by the DNN. Depending on the predicted class, corresponding Shapley values were chosen as scaling factors for PCA coefficients. Within each class, those scaled coefficients were averaged and used for calculating the Shapley-scaled vector lengths associated with each feature. As in the case of a single slice, averaging per feature type, rescaling to the [0, 1] range (cf. Figure S3), and the exclusion of vector lengths below the 75th percentile were performed.

## 3. Results

### 3.1. BPE Classification

**Figure 2:**
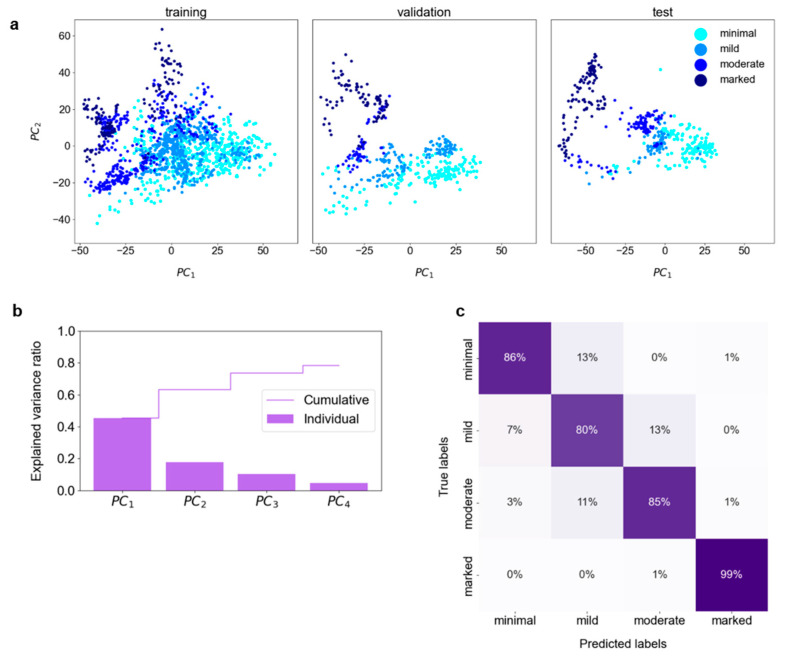
**BPE classification:** (**a**) The Principal Component Analysis (PCA) was performed on the training set, with the validation and test sets transformed accordingly. The first two Principal Components (*PC*s) of the extracted radiomic features present a gradual transition from the lowest to the highest BPE class. (**b**) The cumulative explained variance ratio in the first four *PC*s amounts to 0.78. These *PC*s were used for the training and validation of the deep neural network. (**c**) The confusion matrix for the test set.

Figure 2a depicts the first two *PC*s for each set; a gradual transition from the *minimal* through *mild*, *moderate* to *marked* class is observed. Hyperparameter tuning resulted in the choice of the first four *PC*s as input for DNN training. These *PC*s explained 78% of the variance in the training set (Figure 2b). The DNN modes trained with the best set of hyperparameters achieved an accuracy of 84 ± 2% (*p*-value < 0.00001). The model chosen for detailed analysis classified the test set with 88% accuracy (84–91%, 95% CI; *p*-value < 0.00001), with 93% (85–98%, 95% CI; *p*-value < 0.00001) of misclassifications occurring between the adjacent classes (cf. confusion matrix in Figure 2c). The plot of latent representations of the slices contained in the test set revealed that the misclassified slices have their latent representations mostly in the transition regions between the classes (cf. Figure S4). The model achieved almost perfect agreement with the consensus characterized by κ = 0.83 (0.79–0.87, 95% CI; *p*-value < 0.00001).

The performance of the second DNN model trained with the same parameters but initialized with a different set of random weights and biases is shown in Figures S5 and S6. The model achieved very similar performance with an accuracy of 86% (83–90%, 95% CI; *p*-value < 0.00001) with 92% (84–98%, 95% CI; *p*-value < 0.00001) misclassification involving adjacent classes and κ = 0.82 (0.77–0.86, 95% CI; *p*-value < 0.00001).

The model reported in the main text, used for evaluating the external test set, achieved an accuracy of 72% (69–75% CI; *p*-value < 0.00001) with 55% (49–61%, *p* < 0.00001) misclassifications occurring between adjacent classes and κ = 0.49 (0.44–0.53, 95% CI; *p*-value < 0.00001). The corresponding confusion matrix is presented in Figure S7.

### 3.2. Explainability of the BPE Classification

**Figure 3:**
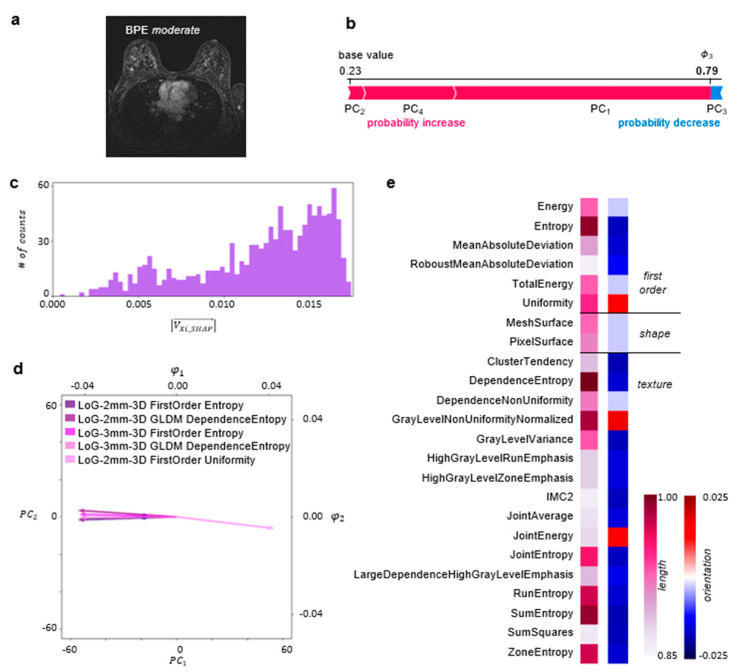
**Local explainability of BPE classification:** (**a**) A single axial 1st subtraction MRI slice classified as BPE-*moderate*. (**b**) A SHAP Kernel Explainer [24] was used to obtain the contribution of each *PC* to the prediction made by the DNN for this slice. These contributions, i.e., Shapley values, are illustrated in the form of a force plot. (**c**) The PCA coefficients were scaled by the corresponding Shapley values, and the Shapley-scaled 4D vector length for each radiomic feature was calculated. The histogram depicts the distribution of those length values. (**d**) The biplot depicts a point describing radiomic features for the MRI slice in the PCA space together with the five longest Shapley-scaled vectors contained in the last bin of the histogram projected onto 2D space. (**e**) The most important radiomic feature types for the DNN prediction, i.e., described by the longest Shapley-scaled vectors. The vector length (1st column) and orientation values (2nd column) are color-coded (details in the Explainability of the BPE Classification section). Abbreviation: Informational Measure of Correlation 2 (IMC2).

An example of the explainability of the DNN predictions for a single MRI slice, contained in a test set and classified by the two radiologists and the DNN as BPE-*moderate*, is illustrated in Figure 3. The DNN classified this slice as BPE-*moderate* with a probability of 0.79. In the presented case, components *PC*_1_, *PC*_2_, and *PC*_4_ contribute to the probability increase, with *PC*_1_ having the highest impact, while *PC*_3_ decreases the probability (cf. Figure 3b).

**Figure 4:**
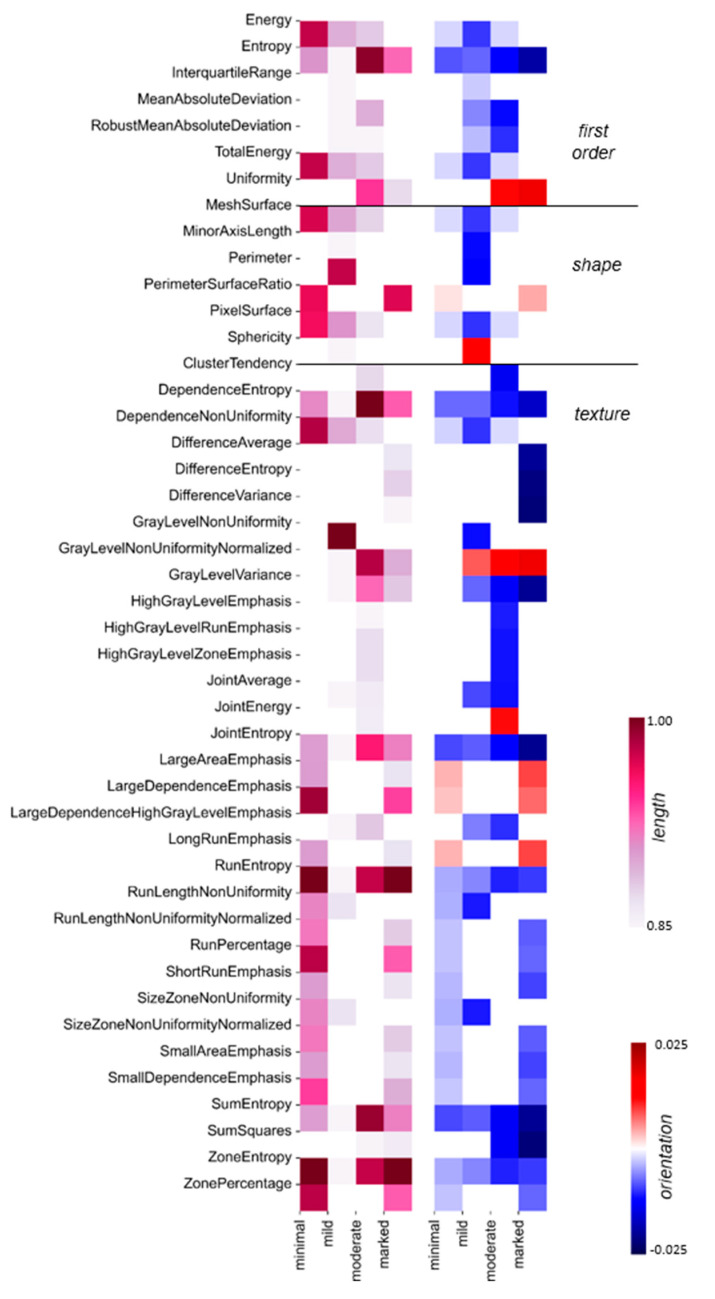
**Global explainability of BPE classification**: For each BPE class, the most important radiomic feature types are depicted: the vector length is color-coded in the 1st heatmap, and the corresponding orientation values are color-coded in the 2nd heatmap. The white places in the diagrams correspond to vector length values below the 75th percentile per BPE class, with the corresponding change values also indicated in white. The color scale is the same as in Figure 3e.

To explain the DNN predictions at the level of radiomic features, the PCA coefficients were scaled by the corresponding Shapley values and served for the calculation of the Shapley-scaled 4D vector’s length and orientation values associated with each radiomic feature following the procedure described in Materials and Methods. Figure 3c illustrates a histogram of the obtained Shapley-scaled vector length values. The five longest vectors, contained in the last bin and projected onto 2D space, are shown in Figure 3d together with a single point corresponding to the representation of radiomic features extracted from the MRI slice in the PCA space. The most impactful radiomic feature types for the DNN prediction are illustrated in Figure 3e. The same analysis was performed for the whole test set, divided into subsets according to the predicted BPE class. The results are illustrated in Figure 4.

The second DNN model trained with the same parameters but initialized with a different set of random weights and biases featured a similar probability for BPE-*moderate* class, i.e., 0.78, for the same single MRI slice, the same features associated with the five longest Shapley-scaled vectors, and the same most impactful feature types had similar vector lengths and orientation values (cf. Figure 3 and Figure S5). Global explainability is characterized by a very similar pattern of feature types with length values above the 75th percentile (cf. Figure 4 and Figure S6).

## 4. Discussion

As outlined in the introduction, this study aimed to achieve two primary objectives: (i) developing an algorithmic pipeline for an accurate BI-RADS-compliant BPE classification, and (ii) ensuring the pipeline’s explainability.

The first goal was realized by developing a dedicated pipeline (Figure 1). BPE classification was reached with an accuracy of 88% (84–91%, 95% CI; *p*-value < 0.00001) (cf. Figure 2c) and the model achieved almost perfect agreement with the consensus: κ = 0.83 (0.79–0.87, 95% CI; *p*-value < 0.00001). While the inherent subjectivity of the visual BPE assessment presents a limitation, the model’s alignment with the gold standard of the consenting expert radiologists, each with more than 5 years of experience in breast imaging, suggests its potential to contribute to the standardization of BPE assessment. Furthermore, in the study by Nam et al. [13], the reported accuracies are 66% and 67% with κ = 0.475 and κ = 0.501 for BPE segmented manually and automatically, respectively. In the study by Borkowski et al. [14], the accuracy amounted to 75% with κ = 0.815 ± 0.13. Although direct comparison between the studies is not possible due to variability in the datasets and utilized approaches, the pipeline presented here achieved promising results.

The second aim was accomplished by combining the PCA coefficients with Shapley values and subsequent calculations of the Shapley-scaled vector length value for each radiomic feature, associated with its importance. 

We have demonstrated the explainability with an example of a single slice classified by the radiologists and the DNN as BPE-*moderate* (cf. Figure 3a). We have shown that the analysis of feature relevance can be performed at the level of radiomic features (cf. Figure 3d) or at the level of radiomic feature types (cf. Figure 3e). Within the radiomic feature level, we have identified the five most impactful radiomic features related to the longest vectors. All of these are associated with the application of the edge-enhancing Laplacian of a Gaussian filter with a coarseness-determining sigma parameter equal to 2 or 3 mm (Figure 3d). The filter application was followed by the calculation of Entropy and Uniformity as well as by GLDM transformation with the subsequent calculation of Dependence Entropy. While entropy-based features constitute a measure of randomness in the gray-level intensity values within region of interest (ROI), Uniformity quantifies the homogeneity of the pixel intensity values. In the presented case, the vectors associated with entropy-based features are oriented in the direction of negative values, while the Uniformity vector is oriented in the direction of positive values in 2D (cf. Figure 3e) as well as in 4D (cf. Supporting Table S2). Noteworthily, the Shapley-scaled vector length value distribution is negatively skewed (cf. Figure 3c), with many vectors having similar length values to the ones described above. For this reason, to gain a more exhaustive analysis of the feature importance, we performed an analysis on the level of radiomic feature types, containing the same radiomic features obtained after the application of various filters and, in some cases, different mathematical transformations (cf. Materials and Methods). For the single MRI slice, the negatively oriented Entropy and the positively oriented Uniformity were the most decisive first-order feature types (cf. Figure 3e). The entropy-based features, i.e., Dependence Entropy, Joint Entropy, Run Entropy, Sum Entropy, and Zone Entropy, were among the most impactful texture feature types, together with Gray Level Non-Uniformity Normalized. The entropy-based feature types are negatively oriented, i.e., towards homogeneity in the intensity values. In contrast, the Gray Level Non-Uniformity Normalized feature type is positively oriented, i.e., towards a non-uniformity of intensity values. This shows that a complex interplay of gray-level quantifying features is decisive for the BPE classification for this slice.

To gain a broader understanding of the feature importance, the explainability approach for the single slice was scaled up for the whole test set in the context of each BPE class (cf. Material and Methods). For 134 slices predicted as BPE-*minimal*, Run Entropy and Zone Entropy feature types were characterized by the longest Shapley-scaled vectors, which were negatively oriented, i.e., towards gray-level homogeneity, which is expected from the visual assessment of the *minimal* enhancement. The BPE-*mild* class was predicted for 105 slices. Here, not only quantitative texture measures of Gray Level Non-Uniformity but also descriptors of the 2D size and shape of the ROI (Perimeter) play a fundamental role in the classification of the slices. From the test set, 105 slices were predicted as BPE-*moderate*. As in the case of a single slice (cf. Figure 3e), there was an interplay between entropy-based feature types oriented towards gray-level homogeneity and Gray Level Non-Uniformity Normalized feature type oriented towards gray-level heterogeneity. For the 104 MRI slices predicted as BPE-*marked*, positively oriented Run Entropy and Zone Entropy had on average the highest impact on these predictions, demonstrating that in the case of the highest enhancement, uniformity in the intensity values also plays a crucial role. Following on from the above discussion, different feature types are decisive for the DNN predictions for each BPE class, reflecting that BPE classification is a task with complex decision boundaries to be found in the feature space.

Noteworthily, our pipeline granting both; high accuracy and explainability did not involve a radiomic feature selection step, commonly included in radiomic-based classification approaches and realized with various algorithms [26,27,28,29]. As demonstrated by A. Demircioğlu, the feature selection step can introduce a bias, which makes identifying radiomic features as biomarker candidates very challenging [28]. In our approach, PCA is performed directly on the extracted radiomic features, with the *PC*s serving as an input to DNN. The number of those *PC*s needs to be chosen based on the explained variance ratio in conjunction with the classifier performance, to find the balance between retaining relevant information for feature explainability and discarding the noise.

Our analysis demonstrates that different feature types are decisive for the DNN predictions of the single BPE classes, which reflects that the BPE classification is a task with complex decision boundaries to be found in the feature space. All three feature families, first-order, shape, and texture, strongly influence the DNN prediction of the BPE class in the case of a single MRI slice and in the case of slices contained in the test set.

The main limitation of the study stems from the limited number of patients, originating from the restricted time of human experts to precisely segment the intricate BPE structures. Additionally, the training data came solely from one scanner at a single institution. The preliminary validation performed with the external dataset of eight examinations acquired with various parameters yielded promising results and underlined the necessity of enriching the training set with data originating from different scanners. Plans are underway to expand the annotations for the EA1141 dataset, which also includes data from GE, Philips, and Merge Healthcare scanners. To ensure a balanced dataset, annotations will be made for 100 patients, considering both the manufacturer and the magnetic field strength of the scanners. Subsequently, an external validation using 50 examinations from our partner institutions will be performed. Due to the time-intensive nature of this task, the retraining and validation processes are expected to be completed by the end of 2024. 

## 5. Conclusions

In summary, we have developed a BI-RADS compliant and explainable pipeline for classifying the contrast uptake in breast tissue during the DCE-MRI breast examination. Our deep learning model achieved an almost perfect agreement with the consensus of breast imaging experts and an accuracy of 88% (84–91%, 95% CI; *p*-value < 0.00001), mainly misclassifying adjacent classes. In the next step, multicenter studies will be performed to validate the pipeline performance for datasets acquired with different imaging protocols and timing of the post-contrast acquisitions.

## Data Availability

The dataset generated and analyzed during the current study is not publicly available due to the confidentiality of the patient data, as stated in the Study Plan approved by the Ethics Committee of Kanton of Zürich. Part of the dataset can be made available from the corresponding author to bona fide researchers for non-commercial purposes on reasonable request.

## Supplementary Materials

The following supporting information can be downloaded at: https://www.mdpi.com/article/10.3390/bioengineering11060556/s1, Supplementary Materials and Methods: MRI image acquisition; Figure S1: The distribution of BPE classes in the dataset as well as in final training, validation, and test sets; Figure S2: Local explainability of the BPE classification; Figure S3: Global explainability of the BPE classification; Figure S4: The 3D latent representations of the slices contained in the test set; Figure S5: Reproducibility of local explainability of the BPE classification; Figure S6: Reproducibility of global explainability of the BPE classification; Figure S7: Evaluation of the model reported in the main text on the external dataset; Table S1: Hyperparameter tuning; Table S2. Shapley-scaled PCA coefficients, vector length, and orientation values associated with radiomic features shown in the biplot in Figure 3d of the main text.
