# Supplementary material for: Explainable Precision Medicine in Breast MRI: A Combined Radiomics and Deep Learning Approach for the Classification of Contrast Agent Uptake
Source: Bioengineering (Basel). 2024 May 31;11(6):556. doi: 10.3390/bioengineering11060556 (PMC11200390; doi:10.3390/bioengineering11060556)
Supplement: Supplementary file 1 [file bioengineering-11-00556-s001.zip › bioengineering-2896252-supplementary.pdf]

Explainable precision medicine in breast MRI: a combined radiomics and deep learning approach for the classification of contrast agent uptake

S. Nowakowska[1]*, K. Borkowski[2], C. Ruppert[1], P. Hejduk[1], A. Ciritsis[1,2], A. Landsmann[1], M. Marcon[1], N. Berger[1†], A. Boss[1‡], C. Rossi[1,2]

[1] Department of Diagnostic and Interventional Radiology, University Hospital Zürich, Rämistrasse 100, 8091 Zürich, Switzerland

[2] b-rayZ AG, Wagistrasse 21, 8952 Schlieren, Switzerland

† Present address: Institut Radiologie, Spital Lachen Oberdorfstrasse 41, 8853 Lachen, Switzerland.

‡Present address: GZO AG Spital Wetzikon, Spitalstrasse 66, 8620 Wetzikon, Switzerland.

* e-mail: S.N. sylwia.nowakowska@usz.ch

SUPPLEMENTRAY MATERIAL

Supplementary Materials and Methods:

Institutional Data: MRI image acquisition. A 3.0 T MRI scanner (MAGNETOM Skyra, Siemens Medical Solution, Erlangen, Germany) with a dedicated 4-channel breast coil was used. The examinations were performed in the prone position and in the axial plane. After the acquisition of an axial T2-weighted short-tau inversion recovery sequence and an axial diffusion-weighted sequence, a dynamic protocol consisting of the acquisition of T1-weighted gradient echo three-dimensional fast low-angle shot sequences (TR/TE 11/ 4.89 ms, flip angle = 10°, voxel = 0.8 mm × 0.8 mm × 1.3 mm) before and after contrast agent administration was acquired in 90 s time intervals. The dose of the contrast agent was adapted to the weight of the patient (0.1 mmol/kg).

Subset of EA1141 dataset: MRI image acquisition.: The examinations were acquired with 3.0 T MRI scanners (Siemens Vario and Skyra) in the axial plane. The DCE sequence was performed with TR =

3.5 – 4.34 ms, TE = 1.31 – 1.97 ms, flip angle 10°, pixel spacing 0.78 mm x 0.78 mm – 0.94 x 0.94 mm, and slice thickness of 1.0 – 2.0 mm.

Software packages: Python 3.8.10 with following libraries: Jupyter Lab 3.3.1, Keras 2.7.0, Matplotlib 3.5.0, Numpy 1.21.4, Pandas 1.3.4, PIL 7.0.0, PyRadiomics 3.0.1, Scikit-learn 1.0.2, Seaborn 0.11.1, SimpleITK 2.1.1, Shapley 1.8.2, Tensoflow 2.7.0.

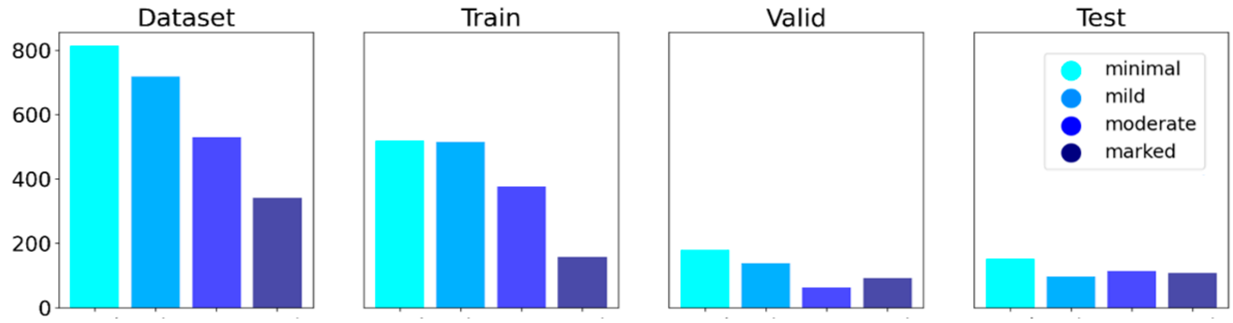

Figure S1 | The distribution of BPE classes in the dataset as well as in final training, validation, and test sets.

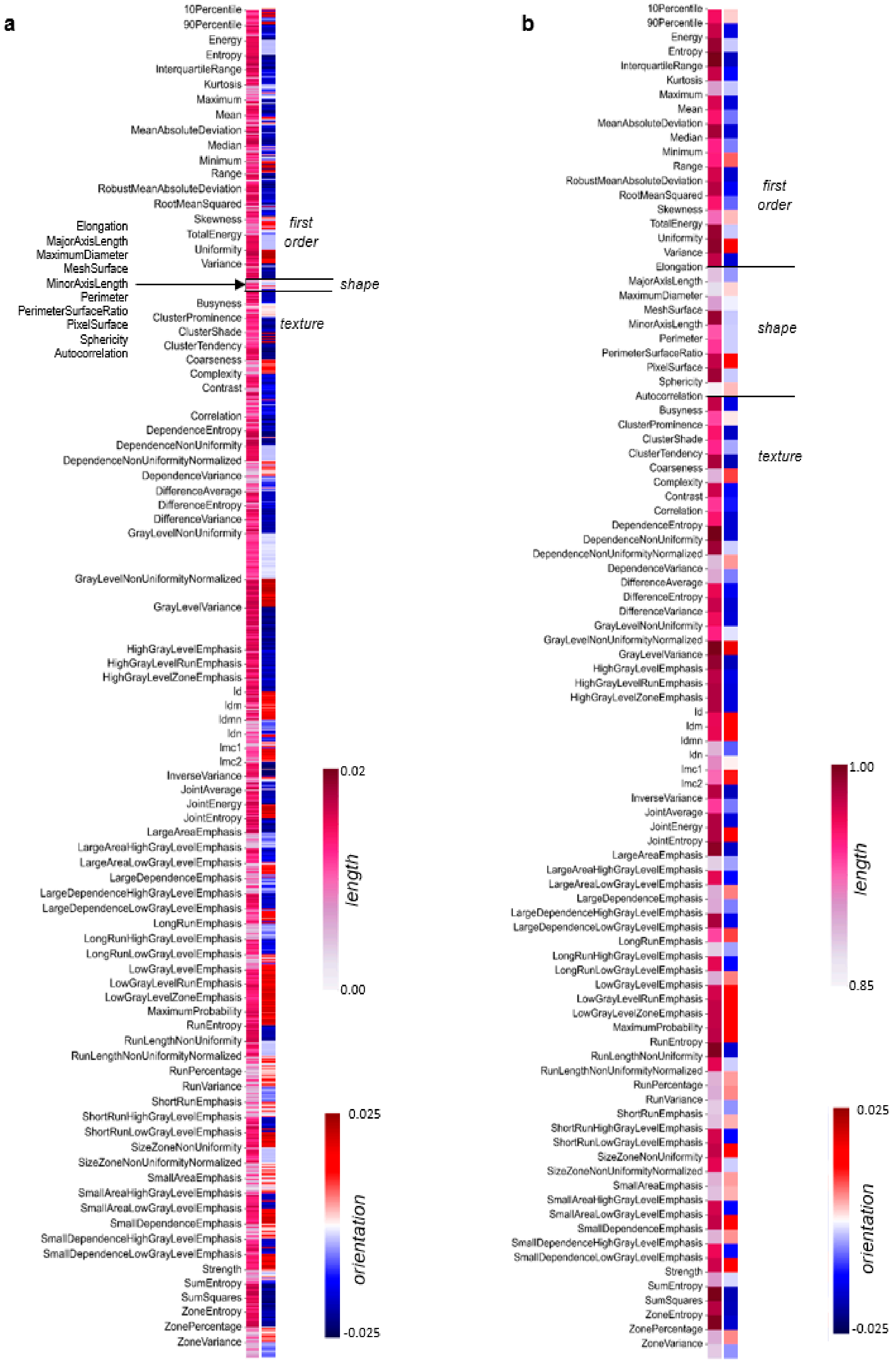
a
10Percentile
90Percentile
Energy
Entropy
InterquartileRange
Kurtosis
Maximum
Mean
MeanAbsoluteDeviation
Median
Minimum
Range
RobustMeanAbsoluteDeviation
RootMeanSquared
Skewness
TotalEnergy
Uniformity
Variance
first order
Elongation
MajorAxisLength
MaximumDiameter
MeshSurface
MinorAxisLength
Perimeter
PerimeterSurfaceRatio
PixelSurface
Sphericity
Autocorrelation
shape
texture
Busyness
ClusterProminence
ClusterShade
ClusterTendency
Coarseness
Complexity
Contrast
Correlation
DependenceEntropy
DependenceNonUniformity
DependenceNonUniformityNormalized
DependenceVariance
DifferenceAverage
DifferenceEntropy
DifferenceVariance
GrayLevelNonUniformity
GrayLevelNonUniformityNormalized
GrayLevelVariance
HighGrayLevelEmphasis
HighGrayLevelRunEmphasis
HighGrayLevelZoneEmphasis
Id
Idm
Idmn
Idn
Imc1
Imc2
InverseVariance
JointAverage
JointEnergy
JointEntropy
LargeAreaEmphasis
LargeAreaHighGrayLevelEmphasis
LargeAreaLowGrayLevelEmphasis
LargeDependenceEmphasis
LargeDependenceHighGrayLevelEmphasis
LargeDependenceLowGrayLevelEmphasis
LongRunEmphasis
LongRunHighGrayLevelEmphasis
LongRunLowGrayLevelEmphasis
LowGrayLevelEmphasis
LowGrayLevelRunEmphasis
LowGrayLevelZoneEmphasis
MaximumProbability
RunEntropy
RunLengthNonUniformity
RunLengthNonUniformityNormalized
RunPercentage
RunVariance
ShortRunEmphasis
ShortRunHighGrayLevelEmphasis
ShortRunLowGrayLevelEmphasis
SizeZoneNonUniformity
SizeZoneNonUniformityNormalized
SmallAreaEmphasis
SmallAreaHighGrayLevelEmphasis
SmallAreaLowGrayLevelEmphasis
SmallDependenceEmphasis
SmallDependenceHighGrayLevelEmphasis
SmallDependenceLowGrayLevelEmphasis
Strength
SumEntropy
SumSquares
ZoneEntropy
ZonePercentage
ZoneVariance
0.02
length
0.00
0.025
orientation
-0.025
b
10Percentile
90Percentile
Energy
Entropy
InterquartileRange
Kurtosis
Maximum
Mean
MeanAbsoluteDeviation
Median
Minimum
Range
RobustMeanAbsoluteDeviation
RootMeanSquared
Skewness
TotalEnergy
Uniformity
Variance
first order
Elongation
MajorAxisLength
MaximumDiameter
MeshSurface
MinorAxisLength
Perimeter
PerimeterSurfaceRatio
PixelSurface
Sphericity
shape
Autocorrelation
Busyness
ClusterProminence
ClusterShade
ClusterTendency
Coarseness
Complexity
Contrast
Correlation
texture
DependenceEntropy
DependenceNonUniformity
DependenceNonUniformityNormalized
DependenceVariance
DifferenceAverage
DifferenceEntropy
DifferenceVariance
GrayLevelNonUniformity
GrayLevelNonUniformityNormalized
GrayLevelVariance
HighGrayLevelEmphasis
HighGrayLevelRunEmphasis
HighGrayLevelZoneEmphasis
Id
Idm
Idmn
Idn
Imc1
Imc2
InverseVariance
JointAverage
JointEnergy
JointEntropy
LargeAreaEmphasis
LargeAreaHighGrayLevelEmphasis
LargeAreaLowGrayLevelEmphasis
LargeDependenceEmphasis
LargeDependenceHighGrayLevelEmphasis
LargeDependenceLowGrayLevelEmphasis
LongRunEmphasis
LongRunHighGrayLevelEmphasis
LongRunLowGrayLevelEmphasis
LowGrayLevelEmphasis
LowGrayLevelRunEmphasis
LowGrayLevelZoneEmphasis
MaximumProbability
RunEntropy
RunLengthNonUniformity
RunLengthNonUniformityNormalized
RunPercentage
RunVariance
ShortRunEmphasis
ShortRunHighGrayLevelEmphasis
ShortRunLowGrayLevelEmphasis
SizeZoneNonUniformity
SizeZoneNonUniformityNormalized
SmallAreaEmphasis
SmallAreaHighGrayLevelEmphasis
SmallAreaLowGrayLevelEmphasis
SmallDependenceEmphasis
SmallDependenceHighGrayLevelEmphasis
SmallDependenceLowGrayLevelEmphasis
Strength
SumEntropy
SumSquares
ZoneEntropy
ZonePercentage
ZoneVariance
1.00
length
0.85
0.025
orientation
-0.025

Figure S2 | Local explainability of the BPE classification: (**a**) The Shapley-scaled vector length values (1st column) with the corresponding orientation values (2nd column) obtained for the single MRI slice classified by the DNN and the radiologists as BPE-*moderate* (displayed in Fig. 3b) sorted per feature type. (**b**) The vector length values averaged per feature type and scaled to the [0, 1] range (1st column) to facilitate the comparability with the global explainability for the BPE-*moderate* class together with the corresponding averaged orientation values (2nd column).

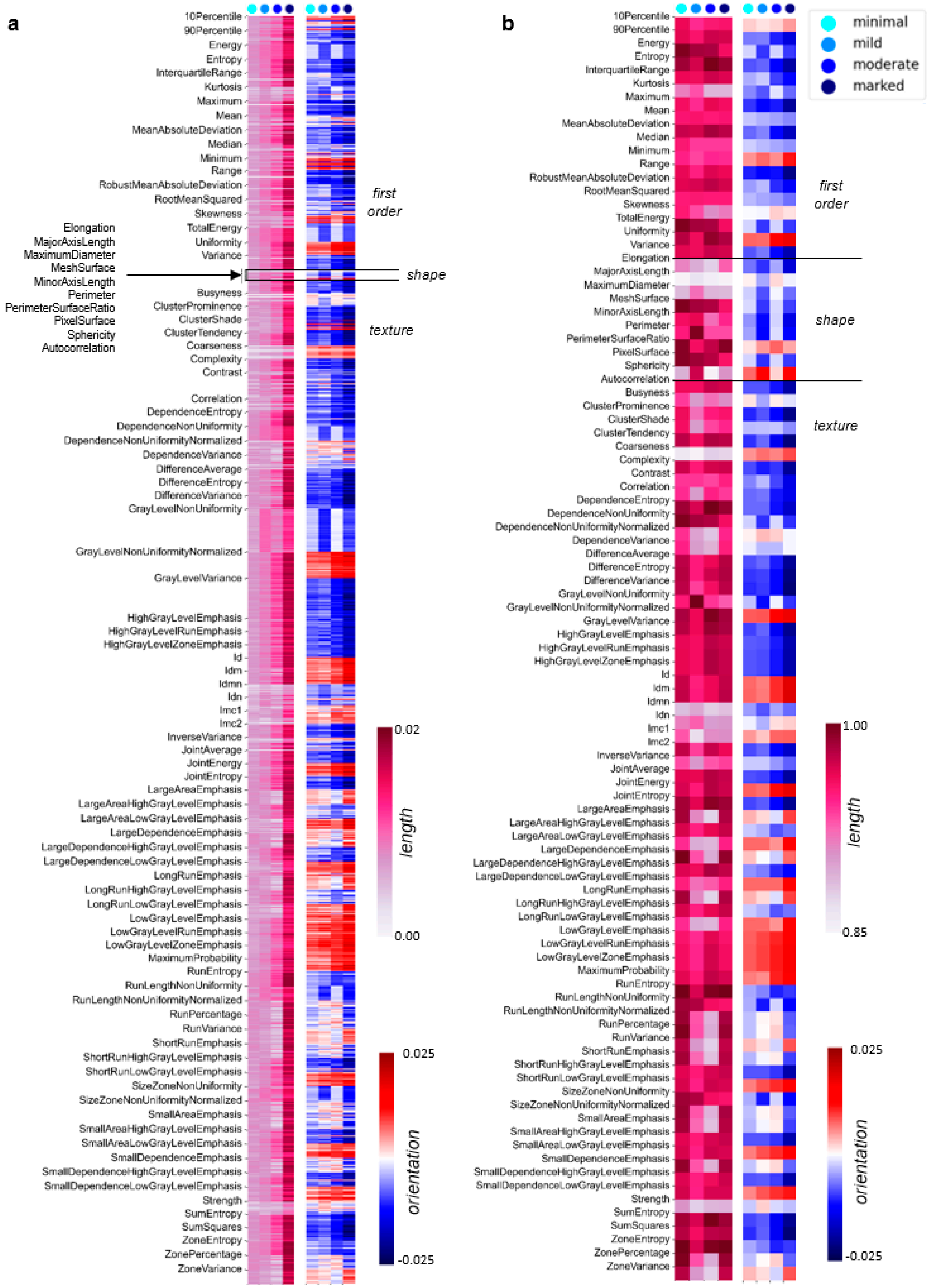
a
b
minimal
mild
moderate
marked
10Percentile
90Percentile
Energy
Entropy
InterquartileRange
Kurtosis
Maximum
Mean
MeanAbsoluteDeviation
Median
Minimum
Range
RobustMeanAbsoluteDeviation
RootMeanSquared
Skewness
TotalEnergy
Uniformity
Variance
Elongation
MajorAxisLength
MaximumDiameter
MeshSurface
MinorAxisLength
Perimeter
PerimeterSurfaceRatio
PixelSurface
Sphericity
Autocorrelation
Busyness
ClusterProminence
ClusterShade
ClusterTendency
Coarseness
Complexity
Contrast
Correlation
DependenceEntropy
DependenceNonUniformity
DependenceNonUniformityNormalized
DependenceVariance
DifferenceAverage
DifferenceEntropy
DifferenceVariance
GrayLevelNonUniformity
GrayLevelNonUniformityNormalized
GrayLevelVariance
HighGrayLevelEmphasis
HighGrayLevelRunEmphasis
HighGrayLevelZoneEmphasis
Id
Idm
Idmn
Idn
Imc1
Imc2
InverseVariance
JointAverage
JointEnergy
JointEntropy
LargeAreaEmphasis
LargeAreaHighGrayLevelEmphasis
LargeAreaLowGrayLevelEmphasis
LargeDependenceEmphasis
LargeDependenceHighGrayLevelEmphasis
LargeDependenceLowGrayLevelEmphasis
LongRunEmphasis
LongRunHighGrayLevelEmphasis
LongRunLowGrayLevelEmphasis
LowGrayLevelEmphasis
LowGrayLevelRunEmphasis
LowGrayLevelZoneEmphasis
MaximumProbability
RunEntropy
RunLengthNonUniformity
RunLengthNonUniformityNormalized
RunPercentage
RunVariance
ShortRunEmphasis
ShortRunHighGrayLevelEmphasis
ShortRunLowGrayLevelEmphasis
SizeZoneNonUniformity
SizeZoneNonUniformityNormalized
SmallAreaEmphasis
SmallAreaHighGrayLevelEmphasis
SmallAreaLowGrayLevelEmphasis
SmallDependenceEmphasis
SmallDependenceHighGrayLevelEmphasis
SmallDependenceLowGrayLevelEmphasis
Strength
SumEntropy
SumSquares
ZoneEntropy
ZonePercentage
ZoneVariance
first order
shape
texture
length
0.02
0.00
1.00
0.85
orientation
0.025
-0.025

Figure S3 | Global explainability of the BPE classification: (**a**) The test dataset was divided into four subsets according to the predicted class. The Shapley values obtained for each slice served as scaling factors for PCA coefficients. These were averaged within each BPE class. The Shapley-scaled vector length values (1st heatmap) and orientation values (2nd heatmap) were subsequently calculated and sorted per feature type. (**b**) The vector length values averaged per feature type and scaled to the [0, 1] range (1st heatmap) to facilitate the comparability with the global explainability for the BPE-*moderate* class together with the corresponding averaged orientation values (2nd heatmap).

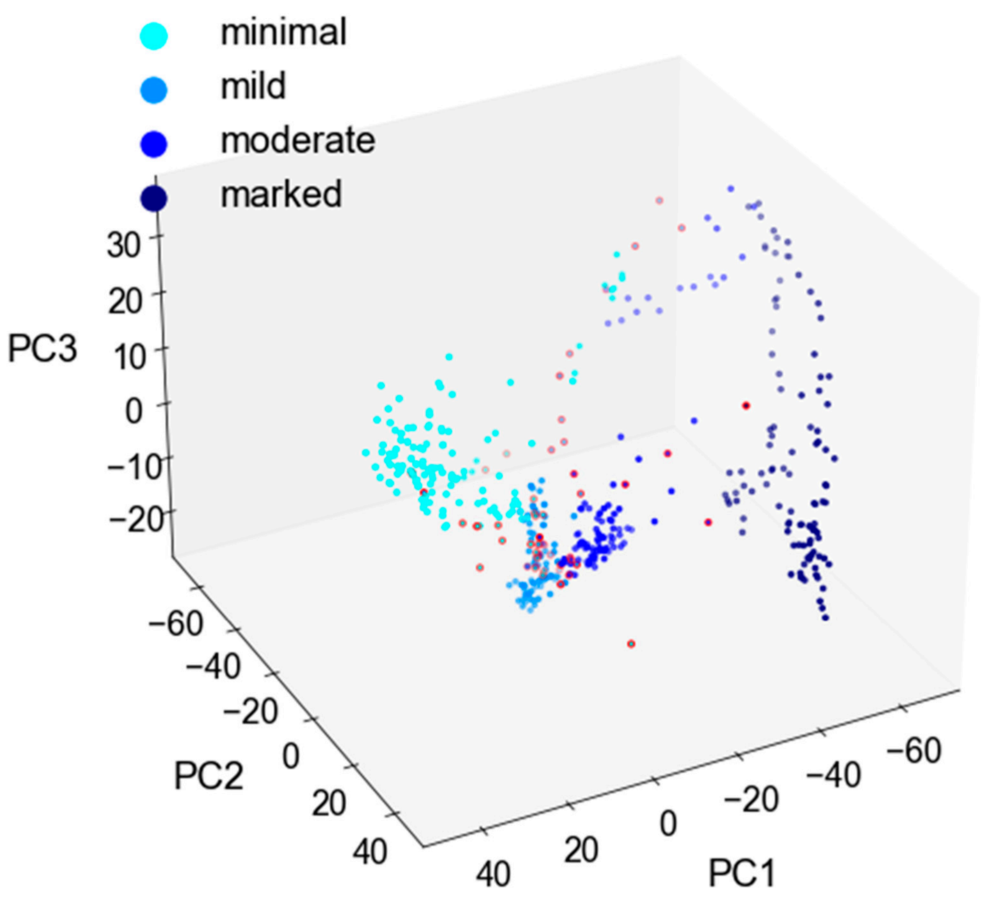

Figure S4 | The 3D latent representations of the slices contained in the test set. The points belonging to misclassified slices are encircled in red.

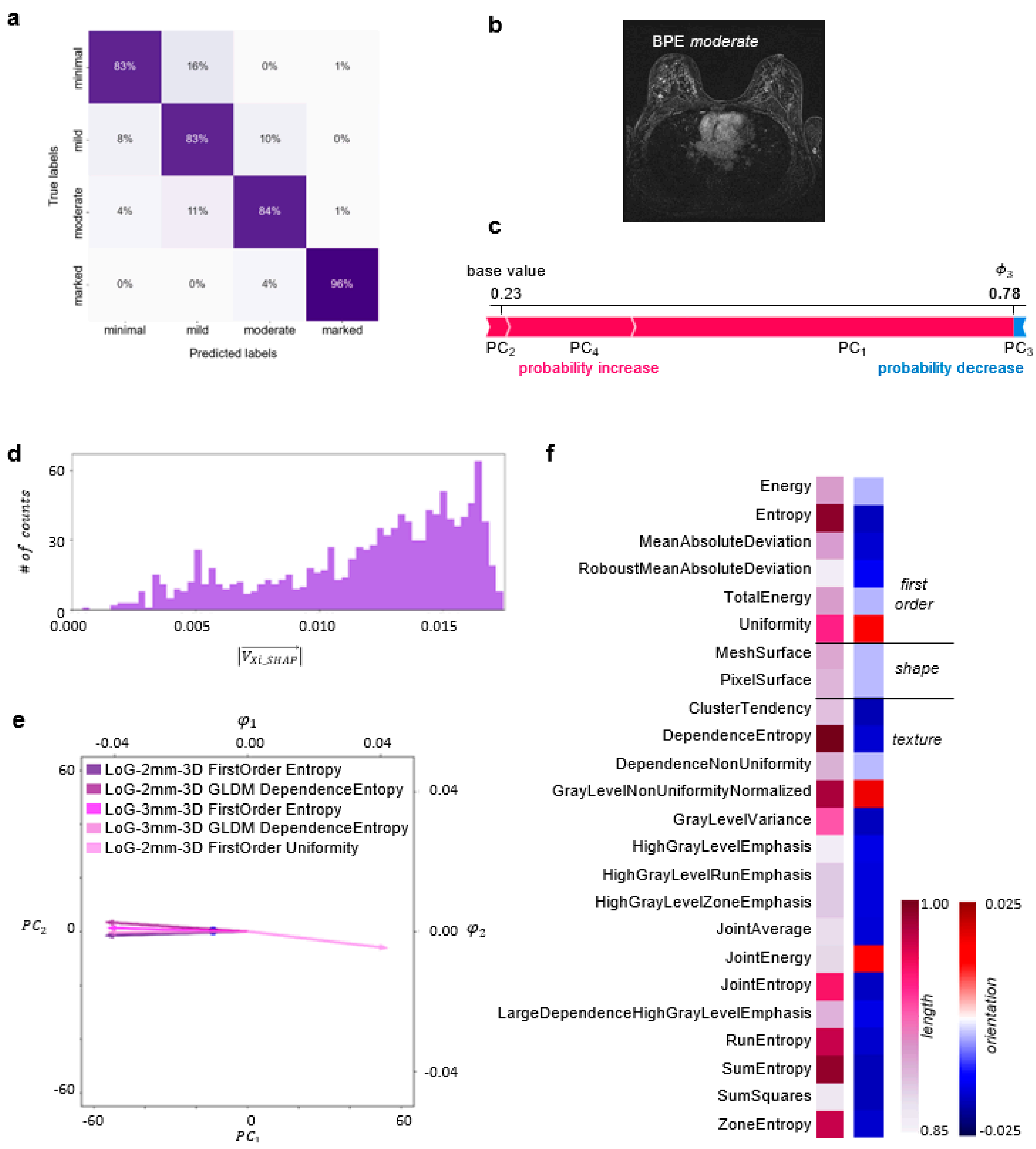

Figure S5 | Reproducibility of local explainability of the BPE classification: Results for a DNN model trained with the same parameters as a model presented in the main text but initialized with a different set of random weights and biases: (**a**) The confusion matrix for the test set: an accuracy of 86% was obtained. (**b** - **f**) Results of the same analysis as shown in Fig. 3 of the main text.

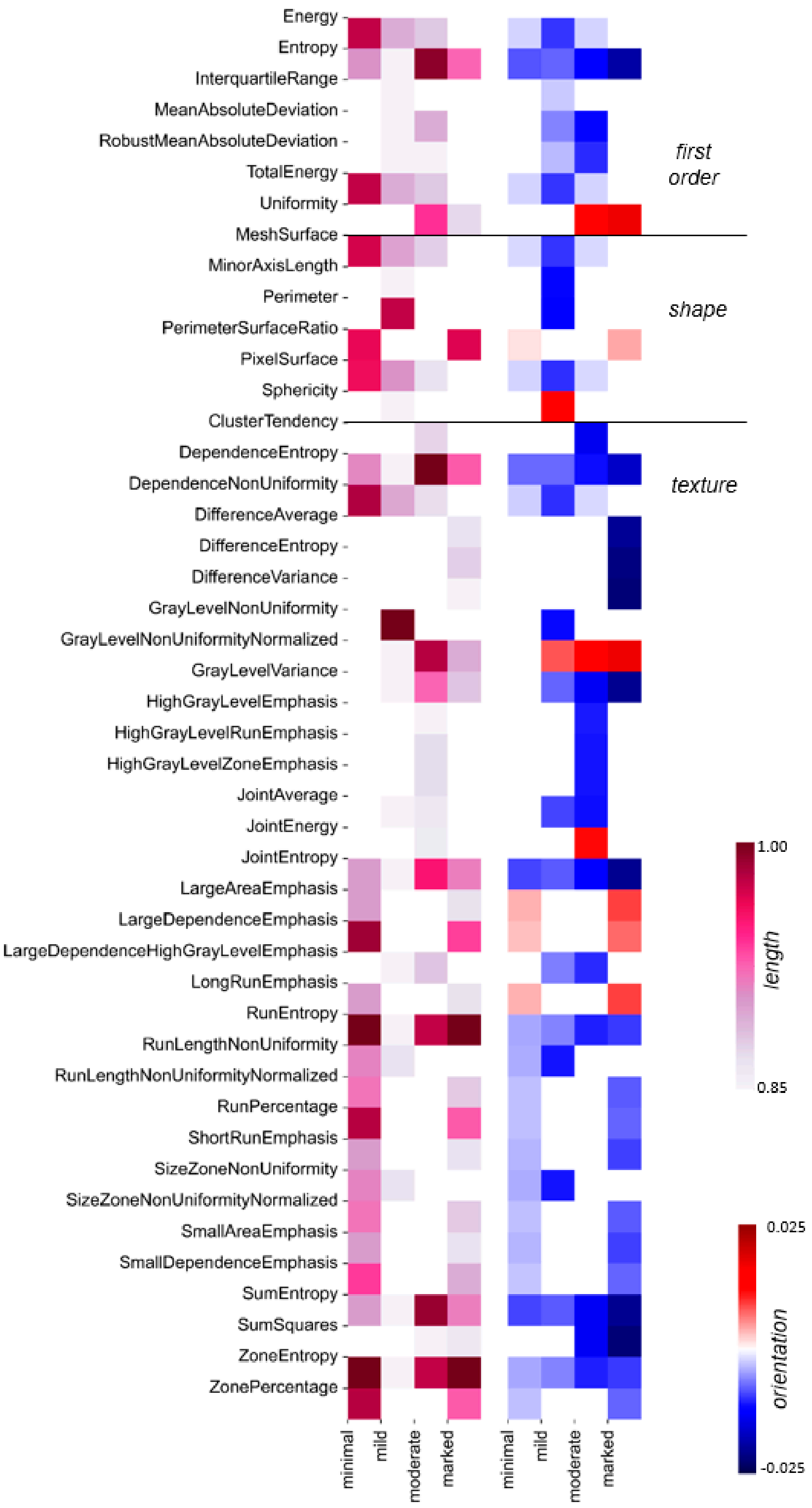

Figure S6 | Reproducibility of global explainability of the BPE classification: Results of the same analysis as shown in Fig. 4 of the main text for a DNN model trained with the same parameters as a model presented in the main text but initialized with a different set of random weights and biases (the reproducibility of the local explainability for this model is shown in Supplementary Fig. S5).

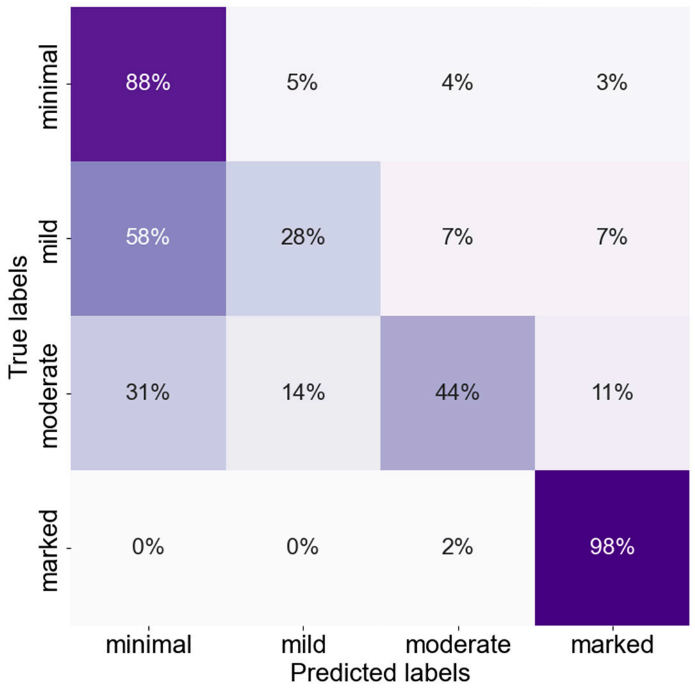

Figure S7 | Evaluation of the model reported in the main text on the external dataset: confusion matrix.

Supplementary Table S1 | Hyperparameter tuning. The hyperparameters yielding best performance are highlighted in gray.

# PCs	# Neurons in dense layers	Learning rate	Dropout rate
2	512, 256, 128, 64	0.0001	0.45
3	512, 256, 128, 64	0.0001	0.45
3	512, 256, 128, 64	0.0001	0.00
3	2048, 1024, 512, 256, 128, 64	0.0001	0.00
4	512, 256, 128, 64	0.0001	0.00
4	512, 256, 128, 64	0.0001	0.45
4	512, 256, 128, 64	0.00001	0.45
4	512, 256, 128, 64	0.0005	0.45

Supplementary Table S2 | Shapley−scaled PCA coefficients, vector length, and orientation values associated with radiomic features shown in the biplot in Figure 3d of the main text.

Filter	Feature family	Feature	PC_1 SHAP	PC_2 SHAP	PC_3 SHAP	PC_4 SHAP	Shapley-scaled vector length	Orientation
LoG-2mm-3D	FirstOrder	Entropy	-1.73E-02	-2.81E-05	-1.68E-04	-1.51E-03	1.74E-02	-1.90E-02
LoG-2mm-3D	GLDM	Dependence Entropy	-1.73E-02	6.27E-05	-1.32E-04	-1.21E-03	1.74E-02	-1.86E-02
LoG-3mm-3D	FirstOrder	Entropy	-1.71E-02	2.45E-05	-1.91E-04	-2.27E-03	1.72E-02	-1.95E-02
LoG-3mm-3D	GLDM	Dependence Entropy	-1.71E-02	-8.06E-06	-1.39E-04	-2.18E-03	1.72E-02	-1.94E-02
LoG-2mm-3D	FirstOrder	Uniformity	1.71E-02	-1.11E-04	1.20E-04	1.44E-03	1.72E-02	1.85E-02
